# Changes in use and outcomes after fibrinogen concentrate insurance coverage for critical obstetrical hemorrhage: a nationwide questionnaire survey in Japan

**DOI:** 10.1038/s41598-024-57244-2

**Published:** 2024-03-20

**Authors:** Masafumi Nii, Tomoaki Oda, Mamoru Morikawa, Yasushi Nakabayashi, Tomoko Adachi, Takao Kobayashi, Atsuo Itakura

**Affiliations:** 1 Department of Obstetrics and Gynecology, Mie University School of Medicine, Tsu, Japan; 2 The Japan Society of Obstetrical, Gynecological and Neonatal Hematology (JSOGNH), Kitakyushu, Japan; 3 Department of Obstetrics and Gynecology, Hamamatsu University School of Medicine, Hamamatsu, Japan; 4 Department of Obstetrics and Gynecology, Kansai Medical University, Osaka, Japan; 5 Department of Obstetrics and Gynecology, Nakabayashi Hospital, Tokyo, Japan; 6 Department of Obstetrics and Gynecology, Aiiku Hospital, Tokyo, Japan; 7 Department of Obstetrics and Gynecology, Hamamatsu Medical Center, Hamamatsu, Japan; 8 Department of Obstetrics and Gynecology, Juntendo University Graduate School of Medicine, Tokyo, Japan

**Keywords:** Critical obstetrical hemorrhage, Fibrinogen concentrates, Public medical insurance coverage, Red blood cell concentrate, Pulmonary edema, Transfusion-induced allergy, Diseases, Medical research

## Abstract

Fibrinogen concentrate (FC) for acquired hypofibrinogenemia associated with critical obstetrical hemorrhage (COH) was covered by public medical insurance in September 2021 in Japan. We aimed to investigate changes in the policy of FC use and its effect on COH after insurance coverage. A primary survey covering September 2020 to August 2021 and a secondary survey covering September 2021 to August 2022 were conducted at 428 higher-level medical facilities. We investigated the policy of FC use in transfusion strategy and the maternal outcomes in COH. Among the hospitals that responded to both surveys, the number of facilities that use FC increased from 51.5 (101/196) to 78.6% (154/196) (*P* < 0.0001). The number of COH cases treated using FC increased from 14.3 to 24.3% (*P* < 0.0001) and that transfused with ≥ 10 units of red blood cells (RBCs) decreased from 36.8 to 29.8% (*P* = 0.001). The incidence of pulmonary edema reduced by 3.7–2.0% (*P* = 0.021), and transfusion-induced allergy by 1.9–0.7% (*P* = 0.008). No changes were observed in the incidence of thromboembolism, arterial embolization, or hysterectomy. The increased use of FC after insurance coverage led to changes in the transfusion strategy, which may be associated with decreases in transfusions of RBCs, pulmonary edema, and transfusion-induced allergies.

## Introduction

Critical obstetrical hemorrhage (COH) is a common cause of maternal mortality worldwide, including in Japan^1–3^. Pathology of COH is generally divided into consumptive coagulopathy associated with placental abruption or amniotic fluid embolism and dilutional coagulopathy associated with uterine atony, placenta previa, placenta accreta spectrum disorders, uterine rupture, uterine inversion, and genital trauma^4–6^. These conditions can induce hypofibrinogenemia and aggravate postpartum hemorrhage^7–10^. Rapid normalization of fibrinogen levels is important for reducing the probability of maternal death due to COH, and fibrinogen concentrate (FC) has been shown to be effective^11–13^. FC is approved in Germany, the Netherlands, Switzerland, and Austria^14–17^. However, until recently, the use of FC for COH was not approved by public medical insurance in Japan. Therefore, administration of fresh-frozen plasma (FFP), which is covered by insurance, was the first-line therapy, along with cryoprecipitate for coagulation factor replacement. The use of FC for acquired hypofibrinogenemia associated with COH has been covered by public medical insurance since September 2021 in Japan^18^.

However, the policy for the use of FC in COH Japan has not yet been investigated, and its efficacy and safety are unknown. In this study, we focused on changes in the FC use policy and its improving effect on the maternal outcome in cases with COH before and after public medical insurance coverage. We hypothesized that introduction of FC with coverage by public medical insurance might improve maternal prognosis in COH along with the increasing number of the cases treated with FC. The aim of our study is to clarify these changes, comparing the responses in nationwide questionnaire surveys conducted during two separate periods.

## Methods

Approximately 800,000 obstetric deliveries per year are performed in approximately 2200 obstetric facilities throughout Japan, and half of all deliveries are managed in approximately 1200 private obstetric clinics operated by only a few obstetricians. Therefore, when an expectant mother at a private obstetric clinic requires further intensive care, such as that for COH, she is transferred to higher-level medical facilities, such as a comprehensive perinatal medical center (PMC), regional PMC, or university hospital. In most cases, comprehensive PMC, regional PMC, and university hospitals are capable to provide advanced care for COH patients and complete the required treatment. Some public hospitals are designated as comprehensive or regional PMC. Among other public hospitals and incorporated hospitals which are not designated as PMCs, patients complicated with COH are also supposed to be transferred to higher-level PMCs or university hospitals, in case that sufficient amounts of blood products for transfusion and/or management of other maternal severe complications such as cerebral hemorrhage and cardiopulmonary dysfunction are not available. We conducted questionnaire surveys during two separate periods in 428 higher-level medical facilities, including 110 comprehensive PMCs, 297 regional PMCs, and 21 university hospitals in Japan. We sent surveillance questionnaires to the personnel in charge of the obstetrical department in each center in paper form via regular mail. The primary survey was conducted from May to June 2022, and the secondary survey from December 2022 to January 2023. The primary survey covered from September 1, 2020 to August 31, 2021, before public health insurance coverage of FC, and the second survey covered from September 1, 2021 to August 31, 2022, after public health insurance coverage of FC. This allowed us to compare the FC use policies and the maternal outcome in cases with COH for a single year before and after public medical insurance coverage of fibrinogen concentrate. Since September 2021, the administration of FC for acquired hypofibrinogenemia associated with COH under public insurance coverage has been limited to these facilities. The Japanese Society of Obstetrics and Gynecology (JSOG) restricts the availability of FC to specific facilities because of concerns regarding the insufficient distribution of FC for congenital hypofibrinogenemia and the requirement of the evaluation of fibrinogen levels prior to administration for potential side effects. The JSOG recommends that, in principle, FC should be administered at fibrinogen levels < 150 mg/dL^19^.

In the present study, COH was defined as a blood loss of 1 L or more during vaginal delivery and 2 L or more during cesarean section, with a fibrinogen level < 150 mg/dL, for the purpose of targeting cases that was expected to be treated with FC. In each survey, we commonly enquired the following five major items about the policy of FC administration for COH, priority of FC in transfusion strategy, availability of fibrinogen level point-of-care testing (POCT) devices, and requests for private obstetric clinics to use fibrinogen level POCT devices and FC. We enquired about the treatment and maternal outcomes in COH, which included the numbers of cases with the following parameters: (1) deliveries, (2) COH, and among COH cases, cases with (3) transfusion of 10 or more units of red blood cells (RBC), (4) transfusion of 15 or more units of FFP, (5) transfusion of one or more packs of cryoprecipitate (in Japan, one pack of cryoprecipitate is produced in-house based on FFP-LR480 (Leukocytes Reduced 480 mL)^20,21^), (6) transfusion of 20 or more units of platelet concentrate (PC) (as apheresis platelets), (7) administration of FC, (8) pulmonary edema, (9) arterial embolization, (10) hysterectomy, (11) thromboembolism, and (12) transfusion-induced allergy. We did not establish our diagnostic criteria for pulmonary edema, thromboembolism, or transfusion-related allergy in the present study. Therefore, we counted the numbers of these cases based on the responses of each institution to the questionnaires. We also surveyed the turnaround time of fibrinogen levels measurement in the central labs in the primary survey and the plans for introducing FC to facilities that responded that they did not have a policy for FC use in the secondary survey. The study from a tertiary obstetric center in Japan reported that medians 8, 14, and 20 units of RBC, FFP, and PC, respectively, were transfused for obstetric hemorrhage patients requiring blood transfusion^22^. It has been also reported that RBC:FFP = 1:1 or higher is recommended for adequate supplementation of coagulation factors without the use of FC^8,9,22^. Based on the reasons demonstrated above, assuming no use of FC, we considered 10 units of RBC, 15 units of FFP, and 20 units of PC, respectively, to be the transfusion doses that could be administered in COH cases in Japan.

Parameters during both periods were compared using the chi-square test or Fisher's exact test, with *P* < 0.05 considered to indicate statistical significance. Statistical analyses were performed using SPSS Statistics 20.0 (International Business Machiens, Armonk, New York, USA). This study was conducted in accordance with the principles of the Declaration of Helsinki and approved by the Clinical Research Ethics Review Committee of Mie University Hospital, Mie, Japan (H2022-007, January 19, 2022). Informed consent was obtained from all participants.

## Results

**Figure 1:**
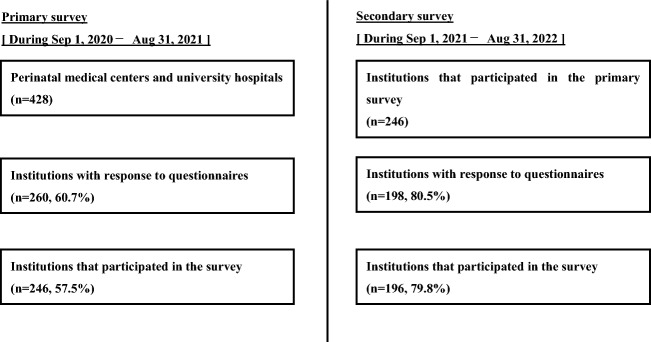
Flowchart of the 2020–2021 and 2021–2022 studies. *Sep September, Aug August.*

Figure 1 shows the numbers of institutions eligible for the primary and secondary surveys. In the primary survey, of the 428 facilities to which questionnaires were sent, 260 (60.7%) responded, of which 246 (57.5%) agreed to participate. In the secondary survey, of the 246 facilities that responded in the primary survey, 198 (80.5%) responded, of which 196 (79.7%) agreed to participate. Table 1 presents the policy of FC administration for COH in the primary survey; 122 facilities (49.6%) had a policy of using FC and 120 (49.8%) did not.

**Table 1:** Policy for the administration of FC in critical obstetrical hemorrhage in the primary survey.

	Comprehensive PMCs (n = 74)	Regional PMCs (n = 165)	University hospitals (n = 7)	Total (n = 246)
Use of FC	42 (56.8%)	76 (46.1%)	4 (57.1%)	122 (49.6%)
No use of FC	32 (43.2%)	86 (52.1%)	2 (28.6%)	120 (48.9%)
Others	0	3 (1.8%)	1 (14.2%)	4 (1.6%)

Data are presented as n (%). The category “Others” included the options that FC was not adopted in the hospital or no policy for FC administration was established.

*PMC* perinatal medical center; *FC* fibrinogen concentrate.

**Figure 2:**
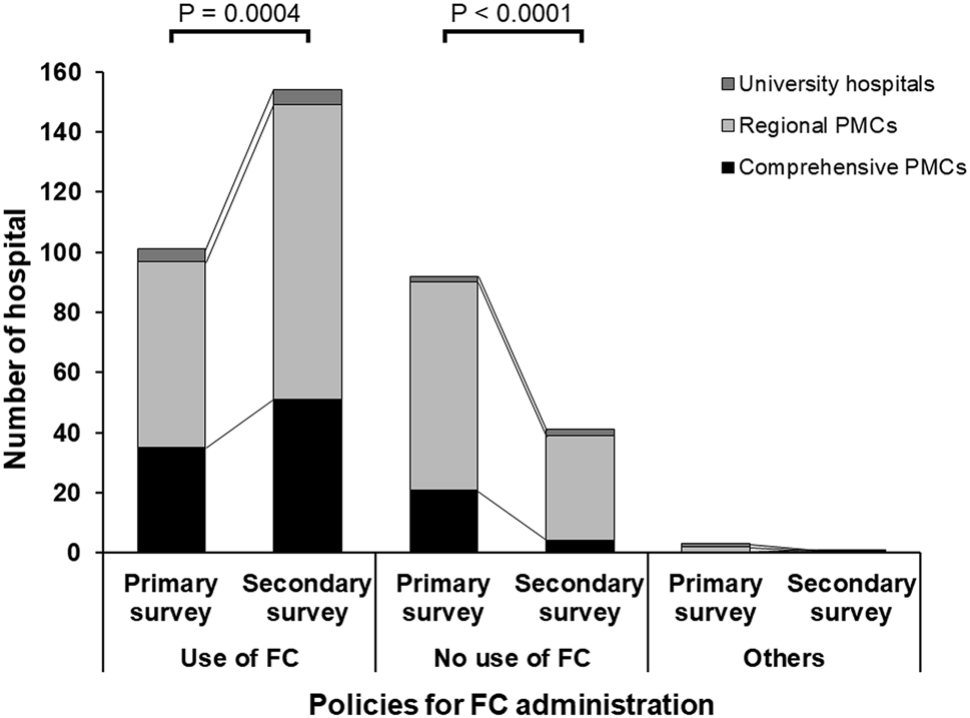
Changes in the policy for FC administration in critical obstetrical hemorrhage between the primary and secondary surveys. The category “Others” included the options that FC was not adopted in the hospital or no policy for FC administration was established. A *P* value < 0.05 was considered significantly different. PMC, perinatal medical center; FC, fibrinogen concentrate.

Figure 2 shows changes in the policy of FC administration for COH between the primary and secondary surveys among the institutions that responded to both surveys. The results of the primary and secondary surveys were compared for the 196 facilities that provided valid responses in the secondary survey. In the primary survey, 101 out of 196 facilities (51.5%) were using FC, while in the secondary survey, the number of facilities using FC increased significantly to 154 out of 196 (78.6%) (*P* < 0.0001). Table 2 shows changes in the policy for prioritization of FC in transfusion strategy for COH. There was no change in the policy regarding the preferential use of FC after insurance coverage. The most common policy was the administration of FFP and/or cryoprecipitate first; FC was administered only when the fibrinogen level dropped to < 150 mg/dL. The percentage of facilities with other policies was similar between before and after public medical insurance coverage of FC.

**Table 2:** Changes in the policy prioritizing FC administration in transfusion strategy for critical obstetrical hemorrhage.

	Primary survey				Secondary survey			
	Comprehensive PMCs (n = 44)	Regional PMCs (n = 77)	University hospitals (n = 4)	Total (n = 125)	Comprehensive PMCs (n = 66)	Regional PMCs (n = 100)	University hospitals (n = 5)	Total (n = 171)
FFP and/or Cryo first	12 (27.3%)	22 (28.6%)	3 (75.0%)	37 (29.6%)	23 (34.8%)	29 (29.0%)	4 (80.0%)	56 (32.7%)
FC first	7 (15.9%)	15 (19.5%)	0	22 (17.6%)	15 (22.7%)	18 (18.0%)	0	33 (19.3%)
FC in non-clottable hemorrhage	9 (20.5%)	13 (16.9%)	0	22 (17.6%)	11 (16.7%)	18 (18.0%)	0	29 (17.0%)
No established policy	5 (11.4%)	14 (18.2%)	1 (25.0%)	20 (16%)	7 (10.6%)	19 (19.0%)	1 (20.0%)	27 (15.8%)
Others	11 (25.0%)	13 (16.9%)	0 (0%)	24 (19.2%)	10 (15.2%)	16 (16.0%)	0 (0%)	26 (15.2%)

Data are presented as n (%). The category “Others” represented the options other than the four policies shown above, including wide variations of policies and situations (e.g., physician's discretion, using both FFP and FC, using FC first in case of prompt FFP administration unavailable, and no adoption of FC). A chi-square test was performed on a 2 × 5 contingency table to evaluate the overall change between the primary and the secondary survey using the sum of the number of each policy answered in the surveys (*P* = 0.94).

*PMC* perinatal medical center; *FFP* fresh frozen plasma; *Cryo* cryoprecipitate; *FC* fibrinogen concentrate.

In the secondary survey, of the 41 facilities that indicated the absence of any policy for FC use, 31 (75.6%) indicated that they might use FC in the future. In contrast, only two facilities (4.9%) indicated that they would not use FC. The remaining 8 facilities have no plans to establish anything (Supplementary Table S1). Table 3 shows the changes in the availability of fibrinogen level POCT devices in PMCs and university hospitals and in requests to private obstetric clinics for using the POCT devices and FC. The availability of fibrinogen level POCT devices did not change after insurance coverage in the PMCs and university hospitals (*P* = 0.81). The rate of requests to private obstetric clinics for the use of FC did not change (*P* = 0.76); however, the rate of requests for the preparation of fibrinogen level POCT devices increased significantly (*P* = 0.036).

**Table 3:** Change in availability of fibrinogen level POCT devices in PMCs and university hospitals, and in requests for private obstetric clinics to use the devices and fibrinogen concentrate.

	Primary survey				Secondary survey				*P* value
	Comprehensive PMCs (n = 56)	Regional PMCs (n = 133)	University hospitals (n = 7)	Total (n = 196)	Comprehensive PMCs (n = 56)	Regional PMCs (n = 133)	University hospitals (n = 7)	Total (n = 196)	
Fib POCT devices available	21 (37.5%)	24 (18.0%)	0	45 (23.0%)	21 (37.5%)	26 (19.5%)	0	47 (24.0%)	0.81
Request to use FC	27 (48.2%)	69 (51.9%)	4 (57.1%)	100 (51.0%)	28 (50.0%)	71 (53.4%)	4 (57.1%)	103 (52.6%)	0.76
Request to use Fib POCT devices	39 (69.6%)	71 (53.4%)	4 (57.1%)	114 (58.2%)	42 (71.4%)	91 (67.7%)	2 (28.6%)	135 (68.9%)	0.036

Data are presented as n (%). A *P* value < 0.05 was considered significantly different.

*Fib* fibrinogen; *POCT* point-of-care testing; *FC* fibrinogen concentrate; *PMC* perinatal medical center.

Table 4 shows the comparison of treatment and maternal outcomes in COH between the primary and secondary surveys. The primary and secondary surveys included 106,907 and 105,432 deliveries, respectively, with 982 (0.9%) and 1020 (1.0%), respectively, cases of COH. The proportion of cases treated with FC increased significantly from 14.3 to 24.3% (*P* < 0.0001). In addition, the proportion of cases in which 10 or more units of RBCs were transfused decreased from 36.8 to 29.8% (*P* = 0.001). However, the proportion of cases in which 15 or more units of FFP, cryoprecipitate, or 20 or more units of PC were transfused did not differ significantly between before and after insurance coverage. Among cases with COH, the incidence of pulmonary edema reduced significantly by about half from 3.7 to 2.0% (*P* = 0.021) and transfusion-induced allergy by about one-third from 1.9 to 0.7% (*P* = 0.008). There was no change in the rates of thromboembolism, arterial embolization, or hysterectomy.

**Table 4:** Comparison of treatment and maternal outcomes in critical obstetrical hemorrhage between primary and secondary surveys.

	Primary survey				Secondary survey				*P* value
	Comprehensive PMCs (n = 56)	Regional PMCs (n = 133)	University hospitals (n = 7)	Total (n = 196)	Comprehensive PMCs (n = 56)	Regional PMCs (n = 133)	University hospitals (n = 7)	Total (n = 196)	
Deliveries	41,688	62,849	2370	106,907	41,432	61,602	2398	105,432	
COH	355	596	31	982 (0.9%)	398	582	40	1020 (1.0%)	0.25
RBC ≥ 10 units	155	193	13	361 (36.8%)	127	166	11	304 (29.8%)	0.001
FFP ≥ 15 units	113	106	17	236 (24.0%)	86	122	12	220 (21.6%)	0.20
Cryo use	34	27	0	61 (6.2%)	25	33	0	58 (5.7%)	0.64
PC ≥ 20 units	65	60	9	134 (13.6%)	63	65	6	134 (13.1%)	0.74
FC use	70	65	5	140 (14.3%)	107	135	6	248 (24.3%)	< 0.0001
Thromboembolism	2	2	0	4 (0.4%)	4	2	2	8 (0.8%)	0.28
Pulmonary edema	22	12	2	36 (3.7%)	8	11	1	20 (2.0%)	0.021
Transfusion-induced allergy	3	12	4	19 (1.9%)	1	6	0	7 (0.7%)	0.008
Arterial embolization	71	70	2	143 (14.6%)	62	71	25	158 (15.5%)	0.57
Hysterectomy	37	39	3	79 (8.0%)	32	31	3	66 (6.5%)	0.39

Data are presented as n or n (%). A *P* value < 0.05 was considered significantly different.

*PMC* perinatal medical center; *COH* critical obstetrical hemorrhage; *RBC* red blood cell; *FFP* fresh frozen plasma; *Cryo* cryoprecipitate; *PC* platelet concentrate; *FC* fibrinogen concentrate.

## Discussion

The number of higher-level medical facilities in Japan with a policy to use FC in transfusion therapy for COH increased after FC was covered by public medical insurance. The number of cases with transfusion of 10 or more units of RBC concentrate, pulmonary edema, and transfusion-induced allergies decreased significantly along with the significant increase in the number of cases with COH that were treated with FC after it was covered by insurance, which indicated that treatment with FC may have contributed to avoid volume expansion and exposure to allergic antigens by RBC transfusion in COH cases.

The insurance coverage of FC has prompted its use for COH in higher-level medical facilities in our country. In this study, the number of facilities that started using FC increased by approximately 30% owing to the newly launched insurance coverage. Notably, of the 41 facilities who responded that they did not intend to use FC in the secondary survey, 31 institutions (75%) considered including FC in their transfusion policy in the future (Supplementary Table S1). When the fibrinogen level falls below 150 mg/dL, the most frequently prioritized method was cryoprecipitate or FFP administration, followed by FC. This could be attributed to the JSOG guideline of replacing coagulation factors with FFP or cryoprecipitate until fibrinogen level dropped below 150 mg/dL^19^ and the application of this policy remained unchanged before and after insurance coverage.

The percentage of higher-level medical facilities with fibrinogen level POCT devices remained at 24.0% in the secondary survey, without an increase after insurance coverage. In contrast, the number of higher-level medical facilities which require primary facilities to use fibrinogen level POCT devices increased significantly by 10%, while that requesting primary facilities to administer FC to COH cases did not change after public insurance coverage. The mean turnaround time of fibrinogen levels measurements in the central laboratory of the higher-level medical facilities surveyed in the primary survey was 32.8 ± 14.3 min (Supplementary Table S2). Although the turnaround time was similar at each type of the higher-level medical facilities, it is expected to take even longer at private obstetric clinics where laboratory technicians and testing equipment are not available. This suggests that more higher-level medical facilities recognized the importance of evaluating fibrinogen levels as soon as possible at the onset of COH to determine the need of maternal transfer, if necessary, rather than infusing FC in private obstetric clinics. Rapid maternal transfer to higher-level medical facilities is a priority in cases with COH which requires multidisciplinary treatment including surgical intervention and blood transfusion, as reported by the Committee to Study Maternal Deaths in Japan^2^. Fibrinogen level POCT devices using dry hematology technology can measure fibrinogen levels in 10–20 min; the measured levels correlate well with the results of the conventional Clauss method for measuring fibrinogen levels^23^. Prompt evaluation of the fibrinogen level using fibrinogen level POCT devices in COH cases with hypofibrinogenemia can support physicians in private obstetric clinics to make their decisions regarding maternal transfer immediately after symptom onset.

Along with the increase in the number of cases in which FC was used for COH at higher-level medical facilities by approximately 1.7-fold because of insurance coverage, the number of patients transfused with 10 or more units of RBC decreased in this study, while there was no change in the use of cryoprecipitate, 15 or more units of FFP, or PC. Rapid normalization of blood fibrinogen levels by FC administration has been reported to reduce the need for RBC and FFP transfusion as well as possibility of blood loss^22,24–26^. The incidence of pulmonary edema and allergies due to blood transfusions decreased after public medical insurance coverage of FC along with the reduction of the number of patients transfused with 10 or more units of RBC. There was no decrease in the case numbers of uterine artery embolization and hysterectomy, however, the fact that cases with thromboembolism, a side effect of FC, did not increase indicates there was no detection of any safety issues regarding the use of FC in this study.

This study has several limitations. First, this was a retrospective questionnaire-based study, and the response rates obtained for both the primary and secondary surveys were 60.7% and 46.3%, respectively, for all facilities. The questionnaires were sent without obligation to the obstetrician-in-chief, and the survey was conducted in two stages over a year. This may have contributed to low response rates. Second, the survey did not examine specific information on individual cases with COH (e.g., primary disease, blood loss, transfusion volume, etc.), making it difficult to analyze a direct causal relationship between FC use and obstetric outcomes. However, the strength of the study is that each parameter was compared at the same center between two periods, thus avoiding the influence of inter-institutional differences in the management of COH.

## Conclusion

Physicians in higher-level medical facilities increased the use of FC in transfusion therapy for COH after public medical insurance coverage of FC, which may be associated with the reduction in patients complicated with pulmonary edema or transfusion-induced allergies along with the decrease in the number of those requiring massive RBC transfusion. Transfusion strategies in combination with FC administration should be further considered for inclusion in facility policies and discussed in patients with COH having hypofibrinogenemia.

### Supplementary Information

Supplementary Information.

## Data Availability

The datasets used and/or analyzed in the current study are available from the corresponding author upon reasonable request.
