# Supplementary material for: Changes in use and outcomes after fibrinogen concentrate insurance coverage for critical obstetrical hemorrhage: a nationwide questionnaire survey in Japan
Source: Sci Rep. 2024 Mar 20;14:6711. doi: 10.1038/s41598-024-57244-2 (PMC10954662; doi:10.1038/s41598-024-57244-2)
Supplement: Supplementary file 1 — Supplementary Information. [file 41598_2024_57244_MOESM1_ESM.docx]

**Supplementary Table S1.** Future plans for the preparation of FC at facilities that responded that they did not use FC according to transfusion policies in the secondary study.

|  | Comprehensive PMCs  (n=4) | Regional PMCs  (n=35) | University hospitals  (n=2) | Total  (n=41) |
| --- | --- | --- | --- | --- |
| Transfusion policy with FC under consideration or will be considered | 3  (75.0%) | 26  (74.3%) | 2  （100％） | 31  (75.6%) |
| No use of FC in any case | 0 | 2  (5.7%) | 0 | 2  (4.9%) |
| No plans to establish anything | 1  (25.0%) | 7  (20.0%) | 0 | 8  (19.5%) |

FC, Fibrinogen concentrate; PMC, perinatal medical center

**Supplementary Table S2.** Turnaround time of fibrinogen levels measurement in the central labs of higher-level medical facilities inquired in the primary study.

|  | Comprehensive PMC  (n=74) | Regional PMC  (n=165) | University hospital  (n=7) | Total  (n=246) |
| --- | --- | --- | --- | --- |
| Turnaround time (min) | 33.0 ± 14.1 | 32.5 ± 14.3 | 38.8 ± 14.6 | 32.8 ± 14.3 |

Turnaround time of fibrinogen levels in the central labs are described as mean ± standard deviation

*PMC* perinatal medical center.
